# Agroecological drivers of aflatoxin contamination in maize-based systems: a review

**DOI:** 10.3389/fpls.2026.1802402

**Published:** 2026-09-18

**Authors:** Winjoy Nyawira Kinyua, Shelmith Wanja Munyiri, John Maina Wagacha, Mary Njoki Mwaura, Patrick Murigu Kamau Njage

**Affiliations:** 1 Department of Plant Science, Chuka University, Chuka, Kenya; 2 Department of Biology, University of Nairobi, Nairobi, Kenya; 3 National Food Institute, Research Group for Genomic Epidemiology and DTU Microbes Initiative, Technical University of Denmark, Kongens Lyngby, Denmark

**Keywords:** aflatoxin mitigation, agronomic practices, aspergillus flavus, biological control, climate stress, food safety, soil microbiome, soil–plant–microbe interactions

## Abstract

Aflatoxin contamination of maize remains a global food safety and public health challenge, particularly because it is a dietary staple food and feed ingredient produced across diverse agroecological zones. Although numerous mitigation strategies have been developed, contamination levels remain variable. This reflects complex interactions among climate, soil properties, crop management practices, plant physiological stress and rhizosphere microbial communities. This review synthesizes evidence on key agroecological drivers of aflatoxin contamination in maize, highlighting climate variability, soil properties, crop and nutrient management, cropping systems, and rhizosphere microbial communities as primary determinants. We examine how these factors influence *Aspergillus* population dynamics, microbial interactions and aflatoxin biosynthesis across contrasting production environments. Evidence synthesized in this review indicates that inconsistent outcomes of existing management including biological control strategies largely arise from insufficient consideration of soil-plant-microbe interactions and spatial heterogeneity. We identify climatic stress, soil properties and crop management strategies as the dominant determinants of aflatoxin risk, acting through biological mediators such as plant physiological status, rhizosphere microbial communities and fungal population. The review argues that effective and scalable aflatoxin mitigation requires integrated, context-specific and systems-based approaches. We conclude that incorporating agroecological heterogeneity into research design, predictive models and management frameworks is essential for improving risk prediction and developing aflatoxin control strategies in maize-based cropping systems.

## 1 Introduction

Aflatoxins are toxic secondary metabolites produced primarily by *Aspergillus flavus* and *Aspergillus parasiticus* and their contamination of maize poses a persistent threat to food safety, human health and livelihoods worldwide (Gemede, 2025; Libamila et al., 2025). Chronic exposure to aflatoxins is linked to hepatocellular carcinoma, immune suppression, stunting in children and reduced livestock productivity (Wolf and Jackson, 1963; Barnes and Butler, 1964; Fouad et al., 2019; Gemede, 2025). Approximately 25% of the world’s food crops are contaminated with mycotoxins annually, with aflatoxins being among the most prevalent and hazardous (Eskola et al., 2020). Aflatoxin contamination occurs across diverse agroecological zones including regions in Africa, Asia, the Americas and increasingly in Europe, where changing climatic conditions are altering fungal distribution and toxin risks (Robens and Cardwell, 2003; Groopman et al., 2014; Medina et al., 2014; Battilani et al., 2016; Jallow et al., 2021; Akullo et al., 2025). The burden of contamination is disproportionately high in Sahara Africa where warm temperatures, periodic stress and limited post-harvest infrastructure coincide with high dependence on maize as a staple (Mutiga et al., 2015; Bandyopadhyay et al., 2019). This situation exacerbates food insecurity by contributing to crop losses, reduced marketability and chronic dietary exposure to aflatoxins, particularly among vulnerable populations (FAO et al., 2021).

A growing body of evidence indicates that aflatoxin contamination is an emergent property of interacting factors, including climate (temperature, rainfall and drought timing), soil physicochemical properties (texture, pH, organic matter and nutrient status), crop management (fertilizer regimes, residue and insect control), cropping system structure (monoculture versus intercropping), and rhizosphere microbial community composition (Battilani et al., 2016; Zhou et al., 2021; Focker et al., 2023; Gonda et al., 2024). Plant stress, particularly drought and heat during the grain-filling stage, can increase kernel susceptibility and trigger aflatoxin biosynthesis in colonizing fungi, while soil properties and native microbiomes modulate inoculum survival, competitive interactions and pathogen establishment (Guo et al., 2008; Zhang et al., 2025). These interactions suggest that aflatoxin risk cannot be adequately understood by considering individual environmental or agronomic factors in isolation; rather, it emerges from their combined effects across the crop–soil–microbiome system.

Previous studies indicate that aflatoxin contamination results from combined effects among climate (temperature, rainfall, drought timing), soil properties (texture, pH, nutrients), crop management, cropping systems and rhizosphere microbial communities (Battilani et al., 2016; Focker et al., 2023; Gonda et al., 2024). Plant stress, particularly drought and heat during grain filling, increases kernel susceptibility and triggers toxin production, while soil and microbial factors modulate inoculum survival and competitive dynamics between toxigenic and atoxigenic strains (Guo et al., 2008; Kebede et al., 2012; Zhang et al., 2025). Although previous research has focused on individual drivers, few integrate agroecological, soil and microbiome interactions to explain variability in contamination and biocontrol efficacy. This review addresses this gap by proposing an integrated agroecological framework for maize-based systems, highlighting how climatic stress, soil and cropping practices are the main drivers of aflatoxin contamination.

Because aflatoxin contamination is influenced by multiple interacting drivers, it is useful to situate these within the broader framework of agricultural productivity, which is commonly categorized into biotic, abiotic and socio-economic factors (Rahman et al., 2022). To maintain a specialized focus, this review is delimited to abiotic stressors that underpin aflatoxin contamination, as these represent critical yet under-represented variables in current modeling frameworks. In this review, we synthesize evidence linking agroecological conditions to aflatoxin risk and to biocontrol efficacy. We first summarize the ecology of aflatoxin-producing *Aspergillus* spp. and known environmental and agronomic drivers of contamination. We then focus on how soil properties, fertilizer regimes and cropping systems especially maize-legume intercropping, modify the soil-plant-microbe link that ultimately governs both toxin production and persistence of biocontrol products. Finally, we propose a conceptual framework for integrating agroecological heterogeneity into predictive risk models and delivery strategies for biological control and identify priority research gaps for improving context-specific mitigation.

## 2 Literature search and selection criteria

A literature search was conducted to identify studies examining agroecological, climatic, soil, microbial and agronomic factors of aflatoxin contamination in maize-based systems. Searches were performed in Web of Science, Scopus, PubMed and Google Scholar, complemented by targeted queries in FAOSTAT, FAOLEX and Codex Alimentarius for regulatory and contextual data. Search terms combined controlled vocabulary and free-text keywords related to aflatoxin (*“aflatoxin*”, “*Aspergillus flavus*”*), maize (*“maize”, “*Zea mays*”*), climate and environmental stressors, soil properties, microbial ecology, crop management, nutrient dynamics, intercropping, biological control, and predictive modelling.* Search strings were adapted for each database but were based on the following core strategy: (Aflatoxin* OR “*Aspergillus flavus*” OR “*Aspergillus parasiticus*”) AND (maize OR corn OR “*Zea mays*”) AND (climate OR temperature OR rainfall OR drought OR “climate change” OR “water activity” OR humidity) AND (soil OR “soil properties” OR “soil microbiome” OR “soil nutrients” OR “soil physicochemical” OR “soil organic matter”) AND (agronomy OR “crop management” OR fertilizer* OR “nitrogen” OR “phosphorus” OR intercropping OR “maize-legume” OR cropping system*) AND (biocontrol OR “biological control” OR “atoxigenic strains” OR “Aflasafe”).

Eligible studies included peer-reviewed articles, reviews, field trials, laboratory studies, modelling work and relevant grey literature published in English, without date restrictions. Papers were screened for relevance to aflatoxin ecology, environmental drivers, soil-plant-microbe interactions, or mitigation strategies. Reference lists of key publications were manually reviewed to capture additional sources. Due to heterogeneity in study designs and metrics, evidence was synthesized narratively and organized thematically around climate stressors, soil and microbiome influences, agronomic practices, biological control and predictive frameworks.

## 3 Overview of maize production

Aflatoxins are among the most significant food safety hazards globally, posing serious threats to human health, agricultural productivity and food security through contamination of major staple crops, particularly in tropical and subtropical regions (Yohannis et al., 2025). Maize (*Zea mays*) is the world’s most widely cultivated cereal due to its critical role in food, feed and industrial applications. Global maize production is dominated by the Americas and Asia, with production of approximately 607.9 and 404.5 million tonnes, respectively. Europe and Africa follow, producing 108.7 and 96.3 million tonnes (**Figure 1**). Maize accounts for a significant portion of global cereal output and feeds millions of people who rely on it as a staple crop (FAOSTAT, 2025).

**Figure 1:**
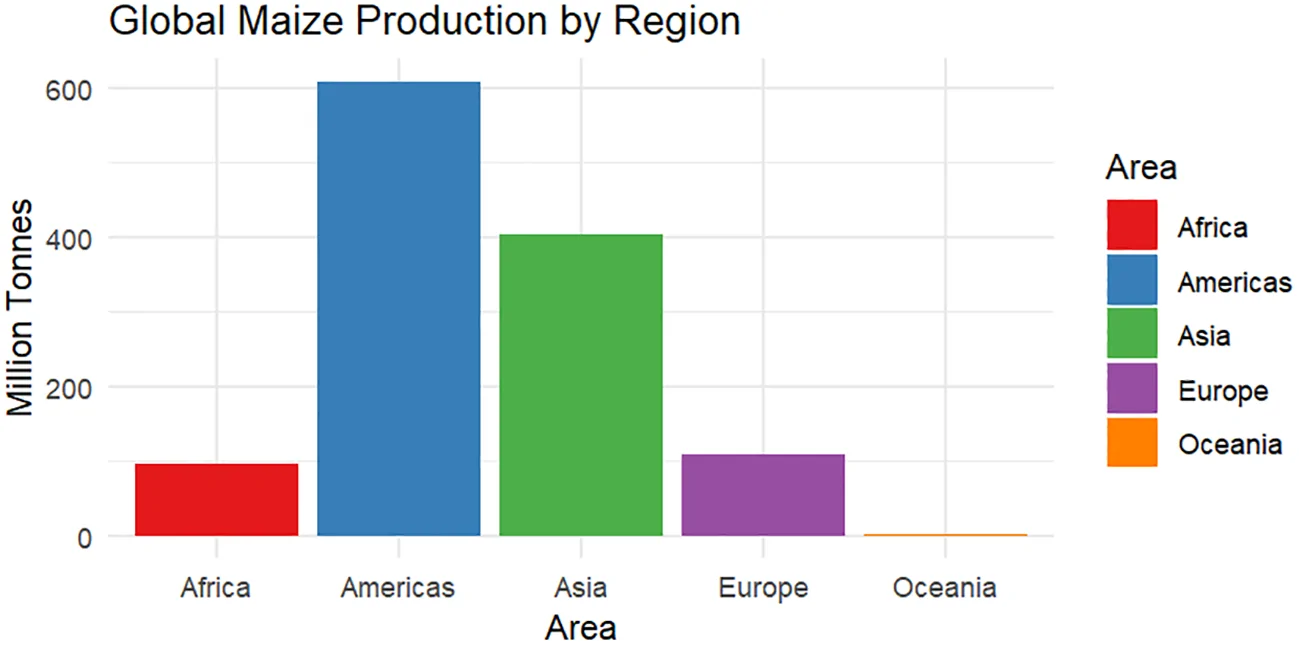
Global maize production by region.

## 4 Global burden and significance of aflatoxins

Aflatoxins are toxic and carcinogenic compounds produced by *Aspergillus* species that commonly contaminate staple crops such as maize, posing serious threats to food safety, public health and causing estimated economic losses of USD 6–18 billion annually (Yohannis et al., 2025). Human and animal exposure through contaminated food and feed is associated primarily with liver toxicity and hepatocellular carcinoma risk (Fouad et al., 2019; Yilmaz and Bag, 2022; Moloi et al., 2024). In Asia, particularly in South and Southeast Asia, warm and humid climates and smallholder-based production and post-harvest practices contribute to high levels of aflatoxin contamination in staple foods such as maize and groundnuts, resulting in chronic dietary exposure and associated health risks in affected populations (Groopman et al., 2014; Nugraha et al., 2018; Jannat et al., 2022).

In the Americas, aflatoxin contamination has been documented in both subsistence and commercial maize systems, with significant implications for food safety regulation and international trade as aflatoxin standards influence maize market dynamics (Wu and Guclu, 2012; Wu, 2015). In Europe, aflatoxin contamination was historically limited but climate change-driven increases in temperature and drought frequency are predicted to expand risk zones in southern and central regions (Battilani et al., 2016). Sub-Saharan Africa remains among the most vulnerable regions due to the convergence of favorable climatic conditions, limited postharvest infrastructure and high dietary reliance on aflatoxin-susceptible crops, representing one component of a broader global aflatoxin challenge (Udomkun et al., 2017; Bandyopadhyay et al., 2019).

Many countries have established regulatory limits for aflatoxins in maize and other foods to reduce exposure risk (Juan et al., 2012; Pickova et al., 2020). The Codex Alimentarius sets limits of 15 µg/kg total aflatoxins in maize for processing and 10 µg/kg in maize flour (FAO, 2020), while regional standards vary: the EU applies stringent limits of 4 µg/kg total aflatoxins (European Union, 2023) and the US FDA allows up to 20 µg/kg in maize for human consumption (Food and Administration, 2010). In Africa, many countries follow Codex guidelines, though enforcement is variable; for example, Kenya limits total aflatoxins to 10 µg/kg (FAOLEX, 2011).

Despite existing regulatory frameworks, surveys indicate that a substantial proportion of maize samples worldwide contain detectable total aflatoxins, highlighting areas of elevated contamination risk. However, even among leading maize-producing countries such as the United States, Argentina, France and parts of Asia, comparable prevalence data remain limited, as studies often emphasize contamination levels and regulatory compliance rather than sample-based occurrence. Notably, even large cross-country assessments, for example by Scott et al. (2026), report occurrence data without consistently providing standardized prevalence percentages across countries. Consequently, the patterns presented in **Table 1** likely reflect both true contamination differences and disparities in surveillance and reporting approaches. The table below summarizes recent studies reporting the percentage of maize samples with detectable total aflatoxins, regardless of regulatory limits.

**Table 1:** Prevalence of total aflatoxins in maize: percentage of contaminated samples across selected countries.

Continent	Country	Production in million tonnes	Highest detectable aflatoxin incidence in the last 10 years	Measurement context	Reference
Africa	Ethiopia	11.7	82.2%	A study on maize for dairy feed	(Tesfaye et al., 2024)
Africa	Kenya	4.0	97%	Multi-city commercial flour surveillance	(Hoffmann et al., 2025)
Africa	Ghana	3.8	35%	Major producing regions across three agro-ecological zones.	(Agbetiameh et al., 2018)
Africa	Malawi	3.0	100%	Country-wide rural survey	(Mwalwayo and Thole, 2016)
South America	Brazil	115.0	61.4%	National mycotoxin survey	(Mallmann, 2020)
South America	Mexico	24.3	88%	Systematic review of occurrence data	(Scott et al., 2026)
Asia	China	295.0	76.7%	Guangxi Province	(Mamo et al., 2021)
Asia	Nepal	3.2	78%	Multi-mycotoxin study on Nepalese maize kernels	(Joshi et al., 2022)
Europe	Italy	5.0	20%	Predictive modeling across Europe	(Battilani et al., 2016)

## 5 Ecology of aflatoxigenic *Aspergillus* species in maize system

### 5.1 *Aspergillus* species: diversity and environmental distribution

Soils support an extraordinarily diverse fungal community, with global species richness estimates in the millions (Tennakoon et al., 2025). Within this diversity, the genus *Aspergillus* is widespread as saprophytes, asymptomatic endophytes and opportunistic plant pathogens across various soil and litter habitats (Nji et al., 2023; Zakaria, 2024). Despite extensive research on aflatoxigenic *Aspergillus* species, the ecological mechanisms governing their environmental distribution and explaining why particular species dominate specific niches remain poorly understood. This limits our ability to predict aflatoxin contamination risk across contrasting agroecological systems.

Key aflatoxigenic species include *A. flavus* and *A. parasiticus*, which are closely related but distinguishable by morphology, isoenzyme patterns and mycotoxin profiles (Klich and Pitt, 1988; Rigó et al., 2002). They thrive in tropical and subtropical regions and have been detected in soils, crop rhizospheres, harvested grains and processed food and feed commodities (Kjærbølling et al., 2020; Mbah et al., 2023). Within *A. flavus*, populations are highly heterogeneous and comprise both toxigenic and atoxigenic strains that differ in aflatoxin biosynthetic capacity, competitiveness and ecological fitness (Ehrlich et al., 2007; Probst et al., 2011).

This decoupling of fungal colonization and toxin production complicates prediction of aflatoxin risk based solely on fungal presence. Vegetative compatibility groups (VCGs) further structure *A. flavus* populations and determine the success of biocontrol strategies based on competitive exclusion. Atoxigenic strains used in commercial biocontrol products belong to VCGs adapted to local agroecological conditions, highlighting the importance of ecological matching between control agents and target environments (Atehnkeng et al., 2014; Bandyopadhyay et al., 2019; Agbetiameh et al., 2020).

### 5.2 Crop contamination patterns in maize and related commodities

Aflatoxins contaminate maize, peanuts, soybeans, chili, tree nuts among other crops (Hassane et al., 2017; Mahato et al., 2019; Balan et al., 2024), whereas wheat, oats, millet, barley, rice, cassava and pulses are generally less susceptible (Bankole et al., 2010; Yohannis et al., 2025). Maize and peanuts are particularly susceptible to aflatoxin contamination (Wagacha and Muthomi, 2008; Njeru et al., 2023). *Aspergillus flavus* persists in soil and plant residues, with overwintering sclerotia serving as the main inoculum. Infection is most critical during maize flowering, facilitated by insect damage and environmental stressors (Battilani et al., 2008; Isakeit et al., 2016). Contamination occurs along the entire value chain, including postharvest stages in crops and animal products (Paterson and Lima, 2010; Kemboi et al., 2020). The “dry chain” approach maintaining low grain moisture that favour fungal activity from harvest to storage significantly reduces aflatoxin risk (Bradford et al., 2020). Implementing integrated field models is critical to inform effective mitigation strategies and enhance food safety.

## 6 Agroecological drivers of aflatoxin contamination

### 6.1 Environmental factors

#### 6.1.1 Climatic drivers of aflatoxin contamination

Environmental factors, particularly temperature and precipitation, are key determinants of fungal proliferation and mycotoxin contamination in crops. However, the relationship between these climatic variables and aflatoxin production is more complex than simple correlation, involving interacting effects that challenge predictive modeling. The temperature-aflatoxin relationship exhibits important non-linearities. Both *A. flavus* and *A. parasiticus* produce maximal aflatoxin levels at approximately 30 °C while synthesis is inhibited at 10 °C (Ogundero, 1987; Kanyi, 2018). Notably, conditions that optimize fungal growth differ from those promoting toxin biosynthesis; warm and humid environments favor colonization but aflatoxin production often intensifies under relatively drier conditions, particularly when crops experience stress (Shabeer et al., 2022; Bunny et al., 2024).

#### 6.1.2 Microenvironmental and mechanistic regulation of aflatoxin contamination

Temperature effects also interact with water activity (aw) and atmospheric composition. Investigations of germination and growth dynamics show minimum aw requirements of 0.82 at 25-30 °C and 0.80 at 37 °C, with colony growth rates increasing with temperature (Pitt and Miscamble, 1995). At the molecular level, regulatory genes *aflR* and *aflS* exhibit temperature-dependent expression, demonstrating a mechanistic basis for temperature-mediated toxin regulation (Gallo et al., 2016; Tian et al., 2021). However, these molecular insights have yet to be translated into practical field-level prediction or intervention strategies. These findings highlight that microenvironmental conditions within grain masses can modulate aflatoxin biosynthesis in ways not captured by ambient temperature and humidity measurements.

Although aflatoxin B_1_ can degrade relatively rapidly in soil under controlled conditions due to microbially mediated processes (Accinelli et al., 2008), field observations indicate that AFB_1_ may persist for extended periods, partly due to its gradual release from infected maize residues. Postharvest maize debris can harbor *Aspergillus flavus* and provide a substrate for continued aflatoxin production, resulting in a dynamic balance between degradation and ongoing replenishment in soil (Accinelli et al., 2008). Furthermore, *A. flavus* is known to thrive under oxygen-rich environments (Gonda et al., 2024), implying that soil aeration which is often shaped by tillage or residue management may further influence fungal activity. This paradox complicates risk assessment, as interventions that limit fungal establishment may not necessarily reduce toxin accumulation.

#### 6.1.3 Field-level evidence of temperature and rainfall interactions in relation to aflatoxin contamination

Field studies in Kenya and Tanzania illustrate the relevance of these interactions between temperature and rainfall in relation to afaltoxin contamination. During the 2013 maize harvest in Kenya and Tanzania, aflatoxin B_1_ incidences ranged from 0 to 100%, with some extreme values reaching 6075 µg/kg-far above the East African (10 µg/kg) and EU (4 µg/kg) regulatory limits (Temba et al., 2021). Elevated temperatures combined with limited rainfall during early crop maturation were identified as key drivers. Diverse topography further complicates predictions: coastal zones experience warm, humid to semi-arid conditions, highlands have cooler sub-temperate climates and intermediate regions exhibit mixed regimes (Ogwang et al., 2014; Temba et al., 2021). Such heterogeneity underscores the importance of fine-scale climate assessment for accurate risk mapping.

#### 6.1.4 Climate change

Climate change is increasingly recognized as a critical driver of aflatoxin contamination in maize systems globally. Changes in temperature regimes, rainfall variability and the frequency of extreme weather events particularly droughts and heatwaves create favorable conditions for *Aspergillus flavus* proliferation and aflatoxin biosynthesis (Medina et al., 2014; Battilani et al., 2016). Elevated temperatures combined with water stress during key crop developmental stages, especially flowering and grain filling, significantly increase host susceptibility and fungal colonization (Kebede et al., 2012; Chauhan et al., 2015).

In temperate regions such as Europe, climate change has contributed to the emergence of aflatoxin contamination in areas previously considered low risk. For example, modeling studies have demonstrated that rising temperatures and increased drought frequency have expanded the geographic range of aflatoxin risk into Southern and Central Europe (Battilani et al., 2016; Fels-Klerx et al., 2016). Similarly, in North and South America, climate variability has been associated with increased aflatoxin occurrence in maize, particularly under drought-prone conditions (Cotty and Jaime-Garcia, 2007; Wu et al., 2014). In tropical and subtropical regions, including parts of Africa and Asia, climate change exacerbates already favorable conditions for aflatoxin contamination by intensifying drought stress and disrupting traditional cropping calendars (Medina et al., 2014; Udomkun et al., 2017). Drought stress not only weakens plant defense mechanisms but also alters kernel composition, making maize more susceptible to fungal invasion and toxin production (Kebede et al., 2012).

Furthermore, climate change influences the ecology and population structure of *Aspergillus* species, potentially favoring more toxigenic strains and altering competitive dynamics between toxigenic and atoxigenic isolates used in biological control (Medina et al., 2017a). These shifts may partly explain the variability in biocontrol efficacy observed across agroecological zones.

Overall, climate change acts as a key abiotic driver that interacts with soil properties and crop management practices to shape aflatoxin risk. Incorporating climate variability into predictive models and management strategies is therefore essential for developing robust, region-specific mitigation approaches.

### 6.2 Soil physicochemical properties

Soil functions as the primary ecological reservoir for *Aspergillus* species and strongly influences their survival, proliferation and aflatoxin-producing potential (Drott et al., 2017). The soil type has been associated with differences in aflatoxin contamination in cropping systems (Bernhoft et al., 2012). Soil texture can also affect the retention of mycotoxins, with clay-rich soils adsorbing toxins more effectively, while sandy soils are more prone to leaching of these compounds (Juraschek et al., 2022). Evidence from peanut production systems further demonstrates that *A. flavus* populations tend to be more abundant and aggressive in clay loam soils compared to sandy loam, which supports lower population densities and reduced infection risk when adequate irrigation is applied (Zhang et al., 2022). However, most of these findings originate from single-crop systems such as peanuts (Dong et al., 2022). Fewer studies have investigated whether similar soil-fungus interactions occur under maize-legume intercropping systems (Mutiga et al., 2015), particularly in semi-arid agro-ecologies. This represents a critical knowledge gap.

Soil pH is another critical determinant of fungal establishment. Most *Aspergillus* species thrive under slightly acidic conditions (pH 5.0-6.0) but some can persist even at extremely low pH levels of approximately 1.5 (Krijgsheld et al., 2012; Kwon-Chung and Sugui, 2013). Because *A. flavus* can tolerate and produce aflatoxins across a broad pH range, variation in soil acidity alone cannot explain observed spatial differences in contamination, highlighting the need to examine interacting environmental and management factors.

Overall, the interaction between soil type, pH, aeration and residue management indicates that no single soil factor independently determines aflatoxin risk. Instead, a complex interplay of physicochemical properties and cropping practices governs fungal establishment and toxin production. Yet, despite extensive work on individual soil variables, few studies have simultaneously evaluated these factors within maize-legume intercropping systems across contrasting agroecological zones.

### 6.3 Soil nutrition

Nitrogen (N) and phosphorus (P) fertilization have demonstrated significant potential in reducing *Aspergillus flavus* colonization and aflatoxin accumulation in maize, though the magnitude and consistency of these effects remain variable across different agroecological contexts. A two-year study in Tanzania tested four fertilizer rates (60 or 120 kg N ha^-1^ with 15 or 30 kg P ha^-^¹) across six maize hybrids at two sites, revealing that optimal N and P application reduced pre-harvest aflatoxin contamination (Manoza et al., 2017; Chen et al., 2019). Similarly, in a study by Nyandi and Pepó (2024) reported that unfertilized controls recorded the highest aflatoxin levels, while N application at 90 and 150 kg ha^-1^ lowered aflatoxin concentrations by 200% and 121%, respectively.

However, despite these promising findings, fertilizer responses were highly genotype- and location-specific. The interaction between nutrient supply, hybrid traits and environmental conditions appears to largely determine the extent to which N and P fertilization suppresses *A. flavus* infection. This variability underscores the need for location-specific fertilizer recommendations rather than blanket approaches. These beneficial effects are attributed to improved plant vigor and stress tolerance, as adequate N and P nutrition strengthens plant defenses, maintains kernel integrity and reduces susceptibility to *Aspergillus* infection during reproductive stages (Manoza et al., 2017). Yet, the mechanistic pathways through which soil nutrient availability influences *Aspergillus* growth, stress tolerance and aflatoxin production remain insufficiently understood. Although studies from Kenya and Zimbabwe suggest that N fertilization improves nutrient uptake and creates soil conditions less favorable to toxigenic fungi (Mutiga et al., 2017; Pasley et al., 2019), little is known about whether N directly influences *Aspergillus* growth dynamics or the expression of aflatoxin biosynthesis genes under field conditions.

In contrast to the relatively extensive work on N and P, evidence on potassium (K) and calcium (Ca) fertilization in relation to aflatoxin mitigation remains scarce. Potassium is essential for enzyme activation, osmoregulation and stress tolerance enabling plants to invest more in physical barriers and repair mechanisms (Marschner, 2011; Oosterhuis et al., 2014), while Ca strengthens cell wall integrity reinforcing physical barriers against pathogen invasion both potentially limiting fungal colonization (Holzmueller et al., 2007; Tripathi et al., 2022). Despite these physiological expectations, there is no direct field evidence linking K or Ca fertilization to reduced *A. flavus* infection or aflatoxin accumulation in maize. Existing conclusions are extrapolated from general plant physiology rather than aflatoxin-specific research. This represents a substantial knowledge gap requiring targeted field trials across diverse agroecological zones-particularly in systems such as maize-legume intercropping, where nutrient dynamics differ significantly from monocultures.

Organic fertilizers and soil amendments are increasingly recognized as effective alternatives or complements to synthetic fertilizers for reducing aflatoxin contamination while enhancing soil health. Organic matter content affects the long-term stability of microbial communities by supporting microbial diversity (Yang et al., 2025). Organic inputs also influence soil physicochemical properties in ways that shift the balance between toxigenic and atoxigenic *Aspergillus* strains. A nine-year multi-omics study reported that manure-based amendments enriched beneficial microbes and metabolites while suppressing soil borne pathogens.

The soil health index improved by 5.2%, 19.3% and 72.6% under 25%, 50% and 100% organic substitution, respectively, when compared with full chemical fertilization (Wei et al., 2025). These results suggest that organic amendments may create soil environments less conducive to the establishment and toxin production of *A. flavus*. However, most of these studies are not maize-specific and very few links microbial shifts directly to aflatoxin outcomes, underscoring the need to integrate soil microbiome analyses with nutrient management trials in maize-based systems.

### 6.4 Rhizosphere and soil microbial communities

Rhizosphere and soil microbial community structure is increasingly recognized as a distinct agroecological driver of aflatoxin contamination in maize systems. The rhizosphere harbors diverse bacteria and fungi whose composition and diversity can significantly affect *Aspergillus* establishment, growth and toxin synthesis. High-throughput sequencing of rhizosphere microbiota has demonstrated that bacterial community composition varies markedly across sites and correlates with aflatoxin levels. Taxa such as Gemmatimonadetes and members of the order Burkholderiales were found to be significantly more abundant in fields with high aflatoxin concentrations. This suggest that specific microbial assemblages may indirectly promote contamination risk by altering niche conditions or microbial interactions in ways that favour *A. flavus* proliferation (Gao et al., 2023).

Rhizosphere and soil microbial communities influencing aflatoxin contamination in maize include toxigenic fungi such as *Aspergillus flavus* and *A. parasiticus*; non-aflatoxigenic *A. flavus*, *Aspergillus niger*, *Trichoderma*, *Penicillium* and *Sarocladium zeae* as fungal biocontrol or antagonistic species; and rhizosphere-associated bacteria including *Pseudomonas*, *Burkholderia*, *Bacillus* and lactic acid bacteria (LAB), which can suppress fungal growth, compete for nutrients, produce antifungal metabolites, or degrade aflatoxins (Palumbo et al., 2007; Mateo et al., 2022; Shabeer et al., 2022; Gao et al., 2023; Wang et al., 2025a).

Microorganisms isolated from maize soil and rhizosphere samples, including *Pseudomonas*, *Burkholderia* and *Bacillus* spp., have shown antagonistic activity against *A. flavus*, limiting fungal growth and indicating that microbial community function can suppress toxin producers when beneficial taxa are present (Palumbo et al., 2007). Evidence from other systems confirms that rhizosphere bacteria and microbial metabolites (e.g., extracellular enzymes and antifungal compounds) can directly inhibit mycotoxigenic fungi, underscoring the mechanistic basis for microbiome-mediated control (Wang et al., 2025a).

The influence of microbial communities interacts strongly with soil physicochemical factors and climatic conditions, as both ecoregion and soil properties shape rhizosphere diversity and composition, which in turn affects maize susceptibility to contamination (Gao et al., 2023). These findings highlight the soil and rhizosphere microbiome as an emergent but under-appreciated driver of aflatoxin risk. It integrates biotic interactions with soil and environmental contexts and that may help explain spatial and temporal variability in contamination outcomes that cannot be accounted for by abiotic factors alone.

### 6.5 Maize-legume intercropping systems

Maize-legume intercropping (MLI) is a multifunctional agroecological practice that may reduce aflatoxin contamination through several interconnected soil-plant-microbe mechanisms rather than a single pathway. It can modify the soil physicochemical environment in ways that may be less favorable for toxigenic *Aspergillus* species. Evidence shows that MLI increases soil pH from acidic monoculture conditions to more neutral ranges (Jalloh et al., 2023), whereas most aflatoxin-producing *Aspergillus* strains grow optimally under more acidic to neutral conditions (pH 5.0-6.0) (Tai et al., 2020).

Legumes enhance nitrogen cycling and plant nutrition through biological nitrogen fixation. Nitrogen transfer to maize in MLI systems occurs at substantially higher rates than in the opposite direction, which theoretically improves plant vigor and reduces stress-related susceptibility to fungal infection (Lai et al., 2022). Maize-legume intercropping also alters the rhizosphere microbial community through diversified root exudates, which recruit distinct microbial taxa (Jiang et al., 2024; Xiao et al., 2023). Studies show clear clustering of bacterial and fungal communities between MLI and monoculture systems (Jalloh et al., 2023), including enrichment of beneficial fungi such as *Sarocladium zeae*, known to produce antifungal metabolites that inhibit *A. flavus* (Poling et al., 2008; Wicklow and Poling, 2009).

In contrast, continuous maize monoculture is widely believed to increase vulnerability to aflatoxigenic fungi due to nutrient depletion, reduced microbial diversity and increased disease pressure (Shu et al., 2024; Li et al., 2025). However, direct comparisons of aflatoxin levels between long-term monoculture and diversified systems remain limited in sub-Saharan Africa.

These mechanistic insights demonstrate how multiple factors likely interact to influence aflatoxin risk in MLI systems. However, their relative contributions remain unclear and no systematic evaluations have been conducted across diverse agroecological zones. Additionally, the long-term stability of these benefits and the possibility of toxigenic *Aspergillus* adapting to MLI-modified environments are still unknown. Together, these mechanisms position maize-legume intercropping as a promising but under-tested agroecological lever for reducing aflatoxin risk and stabilizing biocontrol performance across heterogeneous environments.

### 6.6 Interactions between agronomic, environmental and microbiological factors in aflatoxin contamination

The survival and activity of *Aspergillus* species in maize-based systems are governed by dynamic interactions among soil conditions, climatic variability, cropping systems and field management practices. For example, fertilization does not uniformly reduce aflatoxin contamination; its effectiveness varies with agroecological zone, maize genotype, soil type and prevailing weather during flowering and grain-filling periods when maize is most vulnerable to fungal invasion. Complementary practices such as timely insect control, harvesting at physiological maturity and, in some contexts, fungicide application further influence infection dynamics by limiting fungal entry points and suppressing toxin accumulation (Dövényi-Nagy et al., 2020; Bereziartua et al., 2025; Syraji et al., 2025). Studies across African agroecological zones consistently reveal substantial spatial variability in *A. flavus* distribution and aflatoxin accumulation, reinforcing the need for site-specific nutrient and crop management strategies (Agbetiameh et al., 2018; Massomo, 2020).

Pronounced genetic and ecological diversity exists among both toxigenic and atoxigenic *Aspergillus* strains, with distributions strongly shaped by agroecological gradients and microclimatic conditions (Probst et al., 2010; Monda et al., 2020). However, despite this diversity, key knowledge gaps remain. It is not yet clear how specific agronomic practices such as fertilizer regimes, residue management and maize-legume intercropping interact with soil physicochemical factors to influence the relative abundance and toxigenic potential of *Aspergillus* populations. Few studies have examined these drivers simultaneously, resulting in fragmented insights. Addressing these gaps is crucial for developing sustainable, ecologically grounded aflatoxin mitigation strategies.

Biological control agents (BCAs) are similarly influenced by this complex ecological context. Their establishment, persistence and functional activity depend not only on their genetic traits but also on interactions with indigenous soil microbial communities and with management practices such as intercropping, fertilization and residue retention (Raaijmakers and Mazzola, 2016; Corato, 2020; Xiong et al., 2022). In some cases, strong competition from native microbes or unfavorable soil physicochemical conditions constrains BCA colonization, thereby reducing their effectiveness. These inconsistencies highlight a key limitation in current biocontrol approaches: most fail to account for the broader soil-crop-microbial system in which BCAs operate (Niu et al., 2020). A deeper, systems-level understanding of these interactions is therefore necessary to optimize biological control in real-world field environments.

Emerging evidence also shows that interactions among soil microbial community composition, nutrient status and agronomic practices are further modulated by climatic factors (Dövényi-Nagy et al., 2020; Baazeem et al., 2021; Gao et al., 2023). However, current predictive models often treat climate particularly temperature and precipitation-as standalone determinants of aflatoxin risk, ignoring how climatic stress interacts with soil processes, crop physiology and microbial ecology. As a result, model predictions may fail to capture localized risk patterns or correctly identify high-risk periods within cropping seasons (Medina et al., 2017b; Gachoki et al., 2025). This limitation points to the need for integrative modeling frameworks that incorporate climate-soil-crop-microbiome interactions to improve prediction accuracy and to guide the development of effective, location-specific intervention strategies.

Overall, literature underscores that aflatoxin contamination cannot be explained by individual factors in isolation. Rather, it emerges from multidimensional and context-specific interactions among agronomic practices, environmental conditions and microbial ecology. Understanding and modeling these interactions remains a major research limit with significant implications for designing holistic, scalable and adaptive aflatoxin mitigation strategies.

## 7 Aflatoxin management strategies

Management of aflatoxin contamination involves prevention of fungal growth and inhibition or removal of toxin in maize and other food crops. Aflatoxins are highly stable compounds that resist degradation under a wide range of environmental and processing conditions, making them particularly difficult to eliminate once contamination has occurred. They are thermally stable and can withstand conventional food processing methods such as cooking, roasting and pasteurization, thereby persisting in food and feed products even after treatment (Kabak et al., 2006; Bullerman and Bianchini, 2007). In addition, aflatoxins are chemically stable over a broad pH range and can remain intact during storage, contributing to their long-term persistence in the food chain (Kabak et al., 2006). Contemporary research highlights that integrated approaches combining pre- and post-harvest interventions are the most effective (Liu et al., 2020; Awuchi et al., 2021). Building on the understanding of ecological and environmental drivers of aflatoxin contamination, several approaches have been developed and evaluated. This section critically synthesizes different aflatoxin control strategies,critical assessment of evidence-based mitigation strategies, integration of multiple aflatoxin control strategies and finaly research gaps and challenges in use of available management strategies.

### 7.1 Physical methods

Physical approaches remain central to post-harvest management and include sorting, thermal processing, irradiation, adsorption and emerging non-thermal technologies such as cold plasma and ozone treatment (Peles et al., 2021; Sipos et al., 2021; Gómez-Salazar et al., 2023; Kamano et al., 2025). Cold plasma and low-temperature nitrogen plasma have been shown to reduce *Aspergillus flavus* populations and degrade aflatoxin B_1_ while preserving grain quality (Gómez-Salazar et al., 2023; Kamano et al., 2025; Wang et al., 2025b). Hermetic storage and modified-atmosphere packaging create low-oxygen conditions that suppress fungal growth and limit further aflatoxin accumulation during storage, offering more cost-effective and scalable post-harvest solutions for smallholders, although they do not detoxify grain already contaminated prior to storage (Worku et al., 2022; Khalid et al., 2026).

### 7.2 Chemical methods

Chemical detoxification methods such as ammoniation, fumigation and alkaline treatments can be effective in reducing aflatoxin levels by altering or degrading the toxin molecule (Karlovsky et al., 2016; Adebo et al., 2021). This is achieved by opening the lactone ring and forming fewer toxic products. Alkaline agents can similarly degrade aflatoxins through chemical reactions with their functional groups such as ammonia, bases and acids (Karlovsky et al., 2016; Dib et al., 2022; Sandlin et al., 2025). These approaches offer high detoxification efficiency and are commonly used in animal feed systems, where regulatory frameworks permit their application.

### 7.3 Biological control

Pre-harvest biocontrol strategies reduce aflatoxin contamination by suppressing toxigenic *A. flavus* populations before crop infection and toxin accumulation. A widely implemented approach involves the application of non-aflatoxigenic *A. flavus* strains which outcompete their toxigenic counterparts through currently reported mechanisms such as competitive exclusion and occupation of ecological niches on the crop surface and in the soil. This mechanism effectively limits the colonization and toxin production by aflatoxin-producing strains (Khan et al., 2021; Ortega-Beltran and Bandyopadhyay, 2021, 2023). Other microorganisms, including bacteria (*Bacillus subtilis*, lactic acid bacteria), yeasts and filamentous fungi (*Trichoderma*, *Penicillium* and *Aspergillus niger*), also inhibit aflatoxigenic fungi through nutrient competition, antagonism and secretion of inhibitory metabolites (Ren et al., 2020; Peles et al., 2021; Mateo et al., 2022). While many of these agents show promise under laboratory or semi-field conditions, only non-aflatoxigenic *A. flavus* strains currently have broad field validation and commercial deployment (Moore, 2022).

Globally, several atoxigenic *A. flavus* strains have been developed and commercialized across diverse agroecological regions. In the United States, AF36 and Afla-Guard^®^ (NRRL 21882) have consistently reduced aflatoxin contamination in maize, cottonseed and peanuts under field conditions. In Europe, AF-X1 (Italy) and MyToolBox Af01 (Serbia) have demonstrated significant reductions in maize aflatoxin contamination, confirming the applicability of biocontrol in temperate and Mediterranean systems (Dorner and Lamb, 2006; Moral et al., 2020; Savić et al., 2020; Ouadhene et al., 2024). In Africa, region-specific Aflasafe^®^ formulations tailored to local *A. flavus* populations have achieved >80% reductions in aflatoxin levels in maize, groundnut and sorghum under farmer conditions in West Africa (Senghor et al., 2020). In East Africa, Aflasafe^®^ TZ01 significantly reduced maize aflatoxin contamination in Tanzania, while in Kenya, Aflasafe^®^ KE01, based on native atoxigenic isolates, has been associated with reduced aflatoxin levels and shifts in soil *Aspergillus* communities toward non-toxigenic populations (Migwi et al., 2020; Islam et al., 2021; Fundikira et al., 2025). Biological control using atoxigenic *Aspergillus* strains shows inconsistent performance across locations and seasons, partly due to interactions with native microbial communities and soil physicochemical properties (Focker et al., 2023; Gonda et al., 2024).

### 7.4 Host plant resistance

Host plant resistance represents a sustainable and cost-effective strategy for reducing aflatoxin contamination in maize by limiting fungal infection and subsequent toxin production. Resistance to *Aspergillus flavus* and aflatoxin accumulation is a quantitative trait governed by multiple genetic and physiological factors, including kernel structure, antifungal protein expression and stress tolerance mechanisms (Kebede et al., 2012; Farfan et al., 2015). Several breeding programs have identified maize lines with partial resistance to aflatoxin contamination, characterized by reduced fungal colonization and lower toxin accumulation under both field and storage conditions (Brown et al., 1999; Warburton and Williams, 2014; Okoth et al., 2017). Resistance mechanisms include enhanced physical barriers such as thicker pericarp layers, increased production of pathogenesis-related proteins and the accumulation of secondary metabolites that inhibit fungal growth (Dhakal et al., 2016).

Resistance in maize to aflatoxin accumulation is highly dependent on environmental conditions and agronomic practices. Drought and heat stress, nutrient availability and cropping system structure modulate host susceptibility and fungal virulence, complicating the predictability of resistant varieties under field conditions (Kebede et al., 2012; Warburton and Williams, 2014). Also importantly, resistance is closely linked to tolerance to abiotic stresses, particularly drought and heat which are known to predispose maize to aflatoxin contamination. Drought-tolerant maize varieties have been shown to exhibit reduced aflatoxin levels due to improved plant physiological status and reduced kernel susceptibility (Kebede et al., 2012).

### 7.5 Phytochemical detoxification

Plant-derived phytochemicals are increasingly investigated as natural aflatoxin-detoxifying agents due to their ability to bind, transform or degrade aflatoxin B_1_ (AFB_1_) and inhibit fungal growth (Yang et al., 2024). Essential oils extracted from aromatic plants are among the most extensively studied phytochemicals for aflatoxin control. For example, essential oils from *Thymus vulgaris* (thyme), *Cinnamomum verum* (cinnamon), *Syzygium aromaticum* (clove) and *Origanum vulgare* (oregano) have shown strong inhibitory effects on *A. flavus* growth and aflatoxin B1 production, primarily due to active components such as thymol, eugenol, and carvacrol (Burt, 2004; Kedia et al., 2014). Similarly, *Ocimum basilicum* (basil), *Azadirachta indica* (neem) and extracts from medicinal plants such as *Moringa oleifera* (moringa) have been reported to suppress fungal growth and reduce toxin accumulation in stored grains (Prakash et al., 2015). Ethanolic leaf extracts of *M. oleifera* have been reported to detoxify AFB_1_ completely after 24 h of incubation, while aqueous basil extracts degrade ∼90% of aflatoxins and generate less toxic degradation products, consistent with structural modifications of the toxin molecule upon treatment (Iram et al., 2016; Al-Harrasi et al., 2021).

Botanical extracts such as garlic (*Allium sativum*), turmeric (*Curcuma longa*) and ginger (*Zingiber officinale*) also exhibit antifungal and antiaflatoxigenic properties through mechanisms involving disruption of fungal cell membranes, inhibition of enzymatic pathways involved in toxin biosynthesis and antioxidant activity (Kumar et al., 2017; Mahato et al., 2019). In addition, phenolic-rich plant metabolites have been shown to reduce aflatoxin production by downregulating both regulatory (*aflR*, *aflS*) and structural (*nor-1*, *omt-A*) genes within the aflatoxin biosynthetic cluster in *Aspergillus* species, highlighting their potential as natural modulators of toxin biosynthesis (Hua et al., 1999; El-Desouky, 2025).

### 7.6 Nanotechnology

Recent studies highlight green-synthesized nanomaterials as promising tools for aflatoxin mitigation due to their high surface area and strong toxin-binding capacity (Gelaye and Luo, 2025). Nanoparticles such as silver, zinc oxide (ZnO), titanium dioxide and carbon-based materials interact with aflatoxin B_1_ through electrostatic forces, hydrogen bonding and van der Waals interactions, enabling efficient adsorption, degradation, or neutralization. Green-synthesized nanoparticles have demonstrated up to 50-90% reductions in aflatoxin levels and substantial suppression of *Aspergillus* growth under controlled conditions (Abbasi Pirouz et al., 2021; Gelaye and Luo, 2025; Pena et al., 2025). For example, ZnO nanoparticles significantly reduced *A. flavus* growth and AFB_1_ production in maize media and grain assays, while magnetic graphene oxide nanocomposites effectively adsorbed AFB_1_ (Gelaye and Luo, 2025; Pena et al., 2025). Nano-encapsulated essential oil formulations (e.g., caraway oil in chitosan nanoparticles) also inhibited fungal growth and reduced aflatoxin synthesis by ~93% compared with free oil, suggesting enhanced biocontrol potential (Deepika et al., 2021). Emerging applications propose integration of these nanomaterials into smart packaging and biosensors for real-time aflatoxin monitoring.

### 7.7 Regulatory and monitoring strategies

Regulatory frameworks and monitoring systems are critical components of aflatoxin management, particularly in safeguarding public health and facilitating international trade. Many countries have established maximum allowable limits for aflatoxins in food and feed, typically ranging from 2 to 20 µg/kg depending on the commodity and regulatory authority (Wu, 2015; Eskola et al., 2020). These standards are enforced through routine surveillance, sampling and testing using analytical methods such as high-performance liquid chromatography (HPLC) and enzyme-linked immunosorbent assays (ELISA). Globally, regulatory approaches vary widely, with stricter limits in regions such as the European Union compared to many developing countries, where enforcement capacity may be limited (Eskola et al., 2020). This disparity has significant implications for trade, as contaminated maize may be rejected in international markets, leading to economic losses (Wu, 2015).

Monitoring systems play a crucial role in early detection and risk management. Advances in rapid diagnostic tools, including portable biosensors and immunoassay-based kits, have improved the ability to detect aflatoxins along the value chain (Mahato et al., 2019). In addition, predictive modeling and climate-based risk forecasting are increasingly being integrated into monitoring frameworks to provide early warning systems for aflatoxin outbreaks (Battilani et al., 2016). Despite these advances, challenges remain in harmonizing regulations, improving compliance and strengthening surveillance systems, particularly in low- and middle-income countries (Wu, 2015; Eskola et al., 2020).

### 7.8 Digital and predictive tools

Recent advances in artificial intelligence (AI) have enabled predictive machine-learning models that integrate climate, soil, topographic and satellite-derived variables to map aflatoxin risk in maize production systems (Battilani et al., 2013; Liu et al., 2021). Complementing this approach, the Aflatoxin Risk Early Warning System (A-EWS) applies geospatial AI to forecast pre-harvest contamination hotspots, supporting timely intervention decisions at landscape scale (Gachoki et al., 2025). Recent reviews further confirm that AI and machine-learning frameworks are well suited to capture the non-linear interactions among weather, soil, crop and biological factors that govern mycotoxin contamination, highlighting their growing role in predictive aflatoxin management (Focker et al., 2025; Gachoki et al., 2025).

### 7.9 Critical assessment of integrated evidence-based for aflatoxin mitigation

Evidence across studies indicates that no single mitigation strategy is universally effective. Biological control consistently outperforming other approaches under optimal conditions, while agronomic and post-harvest interventions show more variable and site-dependent outcomes. This variability reflects differences in agroecological conditions, crop responses and microbial interactions that influence intervention performance. Among pre-harvest approaches, biological control using atoxigenic *Aspergillus flavus* strains has consistently demonstrated the highest efficacy under favorable conditions, achieving substantial reductions in aflatoxin levels. Success is strongly influenced by soil type, native microbial competition and timing relative to crop phenology (Khan et al., 2021; Ortega-Beltran and Bandyopadhyay, 2023). However, BCAs are less effective in soils with high microbial antagonism, poor nutrient status, or extreme environmental stress, highlighting the need to integrate microbial ecology with soil management for reliable outcomes.

Soil fertility and nutrient management can indirectly reduce aflatoxin contamination by alleviating plant stress and improving resistance to *A. flavus* infection. Optimized nitrogen and phosphorus application and organic amendments show variable effectiveness across maize genotypes, soil types and rainfall regimes (Manoza et al., 2017; Pasley et al., 2019), underscoring that generalized fertilizer recommendations are insufficient as stand-alone mitigation measures. Similarly, maize-legume intercropping contributes to enhanced nitrogen availability and more diverse rhizosphere microbial communities, which can reduce fungal colonization across agroecologies (Lai et al., 2022; Jalloh et al., 2023). Nevertheless, its benefits are site-dependent and evidence for long-term stability under extreme drought or nutrient-poor soils remains limited.

Plant-derived phytochemicals and essential oils offer natural antifungal and anti-aflatoxigenic effects, particularly in storage systems (Kedia et al., 2014; Yang et al., 2020). While highly effective under controlled conditions, their field application is constrained by volatility, cost, formulation stability and interactions with complex food matrices, limiting their reliability as stand-alone interventions. Post-harvest handling practices including rapid drying, proper storage and insect control are widely recognized as essential across agroecologies. They are generally effective but can be constrained by local infrastructure, access to drying facilities and climatic conditions that promote moisture retention.

Overall, biological control remains the most consistently effective intervention under field conditions, particularly in warm, drought-prone agroecologies where competitive exclusion of toxigenic strains can be sustained. However, its effectiveness depends on compatibility with local fungal populations, soil conditions and appropriate application timing. In contrast, agronomic interventions such as fertilization and intercropping show more variable outcomes across studies, reflecting strong dependence on site-specific factors including soil fertility, crop genotype and environmental stress. Post-harvest strategies remain essential but primarily function to limit further contamination rather than reduce pre-harvest aflatoxin levels.

Collectively, the evidence indicates that no single intervention is universally effective and that its performance depends on the agroecological context in which it is applied. Therefore, the complexity of aflatoxin contamination necessitates the integration of multiple control strategies rather than reliance on single-point interventions (**Figure 2**). Aflatoxin accumulation is driven by interacting environmental, biological and management factors and thus effective mitigation requires a systems-based approach that combines pre-harvest, harvest and postharvest interventions. For instance, the integration of host plant resistance, biological control using atoxigenic *Aspergillus flavus* strains and improved crop management practices such as optimized fertilization and irrigation has been shown to significantly reduce contamination levels compared to individual strategies applied in isolation (Warburton and Williams, 2014; Bandyopadhyay et al., 2016). Similarly, combining proper drying, hermetic storage technologies and sorting with pre-harvest interventions enhances the overall effectiveness of aflatoxin management along the value chain (Torres et al., 2014; Liu et al., 2020). Recent advances highlight the potential of incorporating phytochemicals, improved storage technologies and predictive modeling tools into integrated management frameworks to enhance intervention timing and efficiency (Wu et al., 2014; Mahato et al., 2019).

**Figure 2:**
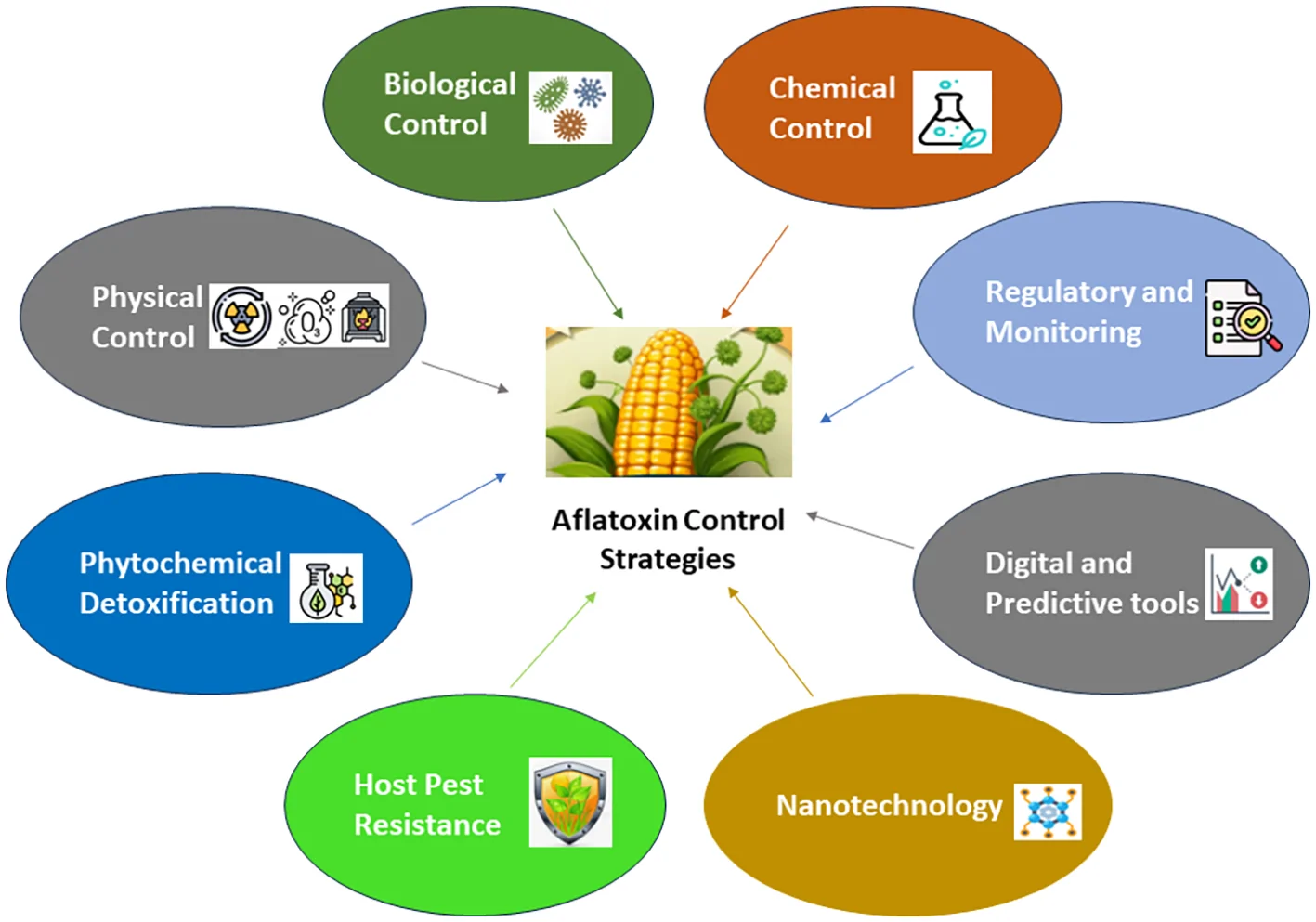
Integrated management strategies for aflatoxin contamination in maize.

However, the success of integrated approaches is highly context-dependent, as interactions among climate variability, soil conditions, cropping systems and microbial communities influence both fungal dynamics and the efficacy of control measures (Battilani et al., 2016; Medina et al., 2017b). Despite these advances, the development of robust, scalable integrated management frameworks remains limited, particularly in resource-constrained settings (Bandyopadhyay et al., 2016; Mahato et al., 2019). This highlights the need for location-specific, climate-smart strategies that account for agroecological heterogeneity.

### 7.10 Research gaps and challenges in use of available management strategies

Despite substantial progress in aflatoxin mitigation strategies, significant research gaps and implementation challenges continue to limit their effectiveness across diverse agroecological and socio-economic contexts (Joint FAO/WHO Expert Committee on Food Additives (JECFA), 2001; Bandyopadhyay et al., 2019). A major limitation across many interventions is the disconnect between laboratory efficacy and field performance. Approaches such as phytochemical treatments, microbial antagonists and emerging nanotechnology-based solutions often demonstrate strong antifungal or detoxification potential under controlled conditions, yet their performance under field conditions remains inconsistent due to environmental variability, complex crop–microbe interactions and heterogeneous storage systems (Atehnkeng et al., 2014; Kumar et al., 2017; Mahato et al., 2019). This highlights the need for multi-location, long-term field validation across representative agroecological zones (Bandyopadhyay et al., 2019).

Post-harvest interventions also exhibit important limitations. Hermetic storage technologies effectively reduce further fungal growth and toxin accumulation but do not eliminate pre-existing aflatoxin contamination (Hell et al., 2010). Advanced detoxification approaches such as irradiation and cold plasma have demonstrated efficacy in experimental settings; however, their adoption is constrained by high costs, technical complexity and limited scalability in smallholder systems (Mahato et al., 2019). Chemical detoxification methods, while effective, are restricted by regulatory limitations, potential toxic residues, impacts on food quality (Kabak et al., 2006; Rushing and Selim, 2019) and high implementation costs, which can make these methods less suitable for smallholder and low-resource contexts (Karlovsky et al., 2016).

In biological control, atoxigenic *Aspergillus flavus* strains have shown effectiveness in reducing aflatoxin contamination; however, their efficacy varies across locations and seasons. This variability is linked to interactions with native fungal populations, soil characteristics and climatic factors, which are not yet fully understood (Bandyopadhyay et al., 2016; Ortega-Beltran et al., 2019). Consequently, predictive frameworks for optimizing biocontrol performance across environments remain underdeveloped. Knowledge gaps remain regarding how soil properties, climatic stress, cropping systems and native microbial communities, resulting in variable performance across locations and seasons (Peles et al., 2021; Bonkoungou et al., 2024).

Host plant resistance is similarly constrained by the quantitative (polygenic) nature of resistance and its strong environmental dependence. Resistance to aflatoxin accumulation is significantly influenced by abiotic stresses such as drought and heat, which exacerbate fungal infection and toxin production (Warburton and Williams, 2014, 2016). The absence of complete resistance underscores the need for integrated breeding strategies that combine aflatoxin resistance with stress tolerance and improved agronomic performance (Warburton and Williams, 2016).

Phytochemical-based interventions face challenges such as volatility, cost, potential impacts on food sensory properties, standardization of extract composition, chemical stability, dosing and field-level validation. Many studies are still at the laboratory stage and do not yet demonstrate consistent performance in complex food matrices or under variable storage and climatic conditions encountered in agricultural supply chains (Gherbawy et al., 2025). Recent research has therefore focused on improving delivery systems, including encapsulation and integration with nanotechnology, to enhance their stability and effectiveness under field and storage conditions. However, these approaches are still emerging and not yet widely implemented in commercial products (Bakkali et al., 2008; Prakash et al., 2015).

Nanoparticles technology experience challenges such as nanoparticle stability, large-scale production, regulatory issues and potential long-term toxicity which still require further investigation (Onyeaka et al., 2022; Gelaye and Luo, 2025). To advance this field, future studies should focus on refining green synthesis processes, enhancing nanoparticle stability and exploring the integration of nanotechnology with biosensors and smart packaging for real-time aflatoxin monitoring (Gelaye and Luo, 2025).

Regulatory and monitoring systems remain unevenly developed, particularly in low- and middle-income countries, where enforcement capacity, infrastructure and surveillance systems are often limited (Wu, 2015; Eskola et al., 2020). Although rapid diagnostic tools and predictive models have improved detection and risk assessment, their routine integration into food safety systems remains limited in resource-constrained settings (Stasiewicz et al., 2017). Digital and predictive tools, including climate-based and machine learning models, have shown potential for forecasting aflatoxin risk; however, their application is constrained by limited availability of high-quality, site-specific data and insufficient validation in smallholder farming systems (Battilani et al., 2016; Bandyopadhyay et al., 2019). This limits their operational utility in regions where aflatoxin contamination is most prevalent.

Limited data availability, inadequate digital infrastructure and challenges with model applicability in low-resource smallholder systems remain key constraints in the use of digital and predictive tools. Artificial intelligence applications often demonstrate high performance in controlled research settings but they have yet to be widely tested or operationalized in smallholder contexts due to gaps in data quality, connectivity and computational capacity (Focker et al., 2025; Ozor et al., 2025). Overall, a key gap is the lack of integrated, context-specific management strategies that combine pre-harvest, post-harvest, regulatory and socio-economic interventions. Future research should prioritize systems-based approaches that emphasize scalability, affordability and adaptation to local agroecological conditions, particularly in smallholder-dominated regions.

## 8 Discussion

This review demonstrates that aflatoxin contamination is an emergent property of interacting climatic, edaphic, agronomic and microbial processes rather than the consequence of any single factor. The apparent inconsistencies reported across studies largely reflect agroecological context, where interactions among climate, soil properties, microbial ecology and crop management determine contamination risk and the effectiveness of mitigation strategies. Climatic influences were consistently non-linear and strongly dependent on crop phenology. Drought and heat stress during flowering and grain filling exerted greater effects on aflatoxin accumulation than seasonal climate averages, suggesting that predictive models should incorporate phenology-specific climatic indicators rather than relying solely on seasonal means (Medina et al., 2014; Battilani et al., 2016).

Beyond climate, soil physicochemical properties, rhizosphere microbial communities and *Aspergillus* population structure emerged as key regulators linking climate and agronomic management with aflatoxin contamination (Drott et al., 2017; Bandyopadhyay et al., 2019; Gao et al., 2023; Gonda et al., 2024). These interactions explain why the effectiveness of fertilizer management, biological control and maize-legume intercropping varies across agroecological zones. Although intercropping consistently improves soil health and microbial diversity, robust field evidence demonstrating sustained reductions in aflatoxin contamination remains limited (Lai et al., 2022; Jalloh et al., 2023). Differences among laboratory, controlled-environment and field studies largely reflect differences in experimental scale rather than conflicting biological mechanisms. Integrating mechanistic and field-based research through coordinated multi-scale studies will improve prediction and support context-specific management strategies (Battilani et al., 2016; Bandyopadhyay et al., 2021).

Overall, the evidence supports a shift from single-factor interventions toward integrated, agroecology-informed management that accounts for interactions among climate, soil, microbial ecology and crop management (Battilani et al., 2016; Medina et al., 2017b). Advances in biological control, predictive modelling and artificial intelligence provide opportunities to improve location-specific risk prediction when integrated with sound agronomic and post-harvest practices (Focker et al., 2025; Gachoki et al., 2025). The conceptual framework proposed in **Figure 3** provides a foundation for developing predictive decision-support systems and highlights the need for multidisciplinary, multi-scale research integrating climate, soil, microbiome and crop management data to strengthen sustainable aflatoxin management.

**Figure 3:**
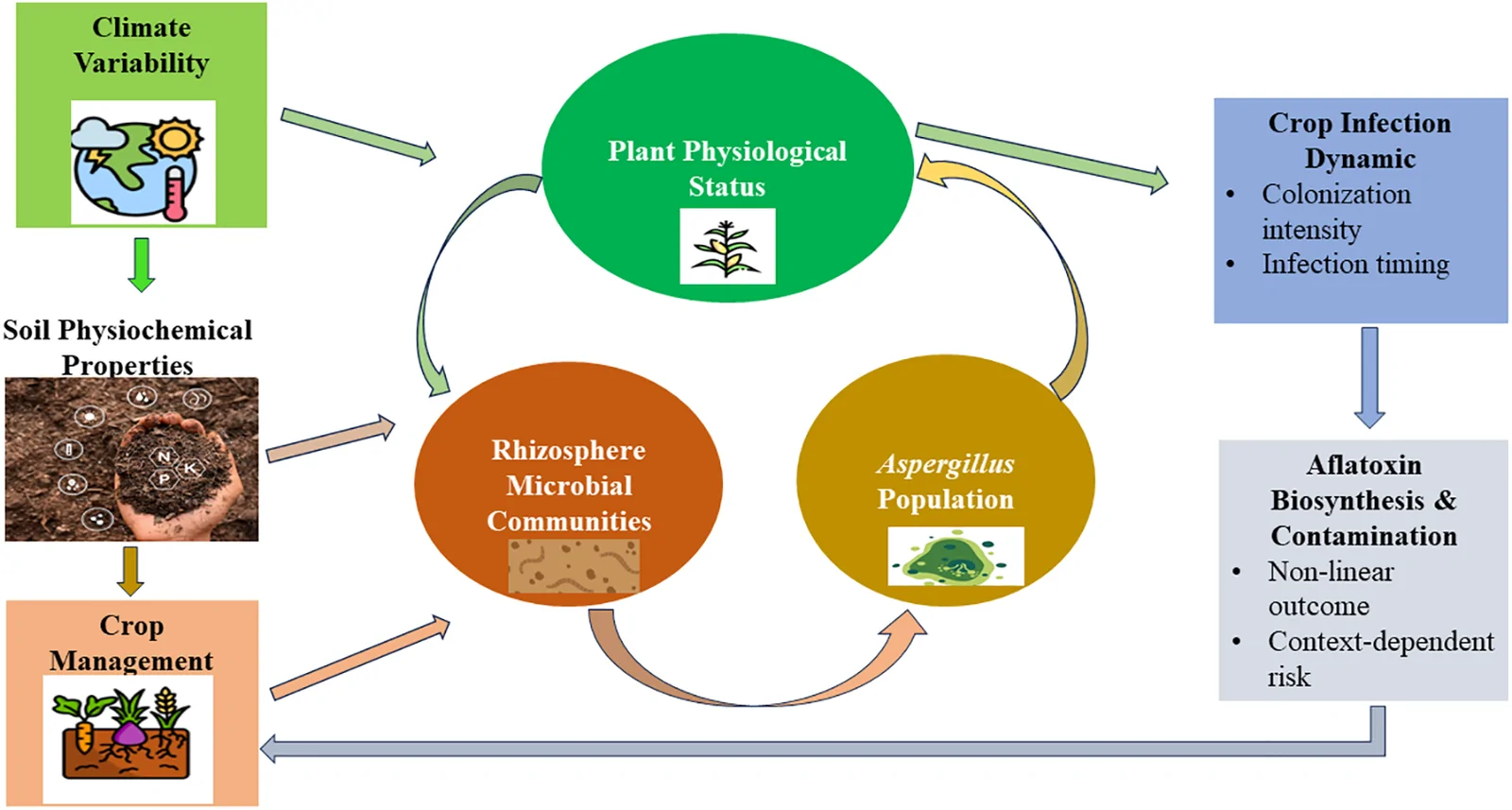
Conceptual framework illustrating agroecological drivers and biological mediators of aflatoxin contamination in maize-based cropping systems.

## 9 Conclusions and future perspectives

Aflatoxin contamination in maize-based systems is driven by complex interactions among climatic stress, soil physicochemical properties, crop management practices and microbial communities rather than by individual risk factors. The evidence synthesized in this review demonstrates that these interacting drivers shape *Aspergillus* population dynamics, plant stress responses and aflatoxin biosynthesis, explaining the variability in contamination observed across agroecological zones. Consequently, effective aflatoxin management requires integrated, agroecology-informed strategies that combine biological, agronomic and post-harvest interventions tailored to local environmental conditions rather than relying on single-factor approaches.

Future progress will depend on developing predictive, systems-based frameworks that integrate climate, soil, agronomic and microbiome data to improve aflatoxin risk prediction and guide targeted interventions. Incorporating soil health indicators, microbial community dynamics and advances in machine learning into early-warning and decision-support systems offers promising opportunities for more precise, climate-smart and sustainable aflatoxin management across diverse maize production systems.
